# Multiple Brain Abscesses due to *Streptococcus anginosus*: Prediction of Mortality by an Imaging Severity Index Score

**DOI:** 10.1155/2016/7040352

**Published:** 2016-03-01

**Authors:** K. O. Kragha

**Affiliations:** Department of Radiology, University of Louisville, 530 S. S. Jackson Street, CCB C07, Louisville, KY 40202, USA

## Abstract

An elderly patient with altered mental status, brain abscesses, ventriculitis, and empyemas died of septic shock and brain abscesses secondary to* Streptococcus anginosus* despite aggressive treatment. An imaging severity index score with a better prognostic value than the Glasgow coma scale predicted mortality in this patient.

## 1. Introduction

Brain abscess is a local infection within the brain parenchyma that starts as cerebritis and then becomes collection of pus with well vascularized capsule. There are four routes of brain infection: contiguous infection, hematogenous spread to the brain from distant infection, direct implant (trauma or neurosurgery), and peripheral nerves. Brain contiguous infection is usually by odontogenic abscesses, sinusitis, and otomastoiditis. Hematogenous brain abscess is usually in the distribution of the middle cerebral artery, near the gray-white matter junction, and multiple. Brain abscess has an estimated incidence of 0.13–0.9 per 100,000 person-years and mortality of up to 20–70%, mean age of 35–37 years, and a male to female ratio of 1.3 : 1 to 3.0 : 1. Brain abscess may cause long-term neurological deficits or be life threatening. Brain abscess is usually caused by multiple bacterial, fungi, or parasites with* Streptococcus* which is the most common organism. Other less common microbes are* Listeria monocytogenes*, staphylococci, Gram-negative bacilli, or* Haemophilus influenza*. Up to about 30% of brain abscesses are cryptogenic. Brain abscess mortality has decreased over the past few decades due to improved diagnosis and improved antibiotic therapy (cf.  Table 1) [1–7].

This case presents the clinical, imaging, and imaging severity index score features and treatment of multiple intracranial abscesses due to* Streptococcus anginosus* that are important for its management and prediction of the outcome.

## 2. Case Report

This is a 61-year-old female who was admitted with altered mental status. The patient had 1-day history of confusion and hemoptysis that began about 4 days prior to admission. Patient was very agitated, unable to communicate, kept her eyes clenched throughout the entire examination, and would not allow examiners to open her eyes. They were pried open briefly and pupils appeared to be reactive. However, pupillary reaction could not be assessed secondary to her agitation and keeping her eyes clenched tight. The patient moved all her extremities well and tried to climb out of bed. Her right patellar and biceps reflexes were 2/4; reflexes on the left were diminished.

**Figure 1:**
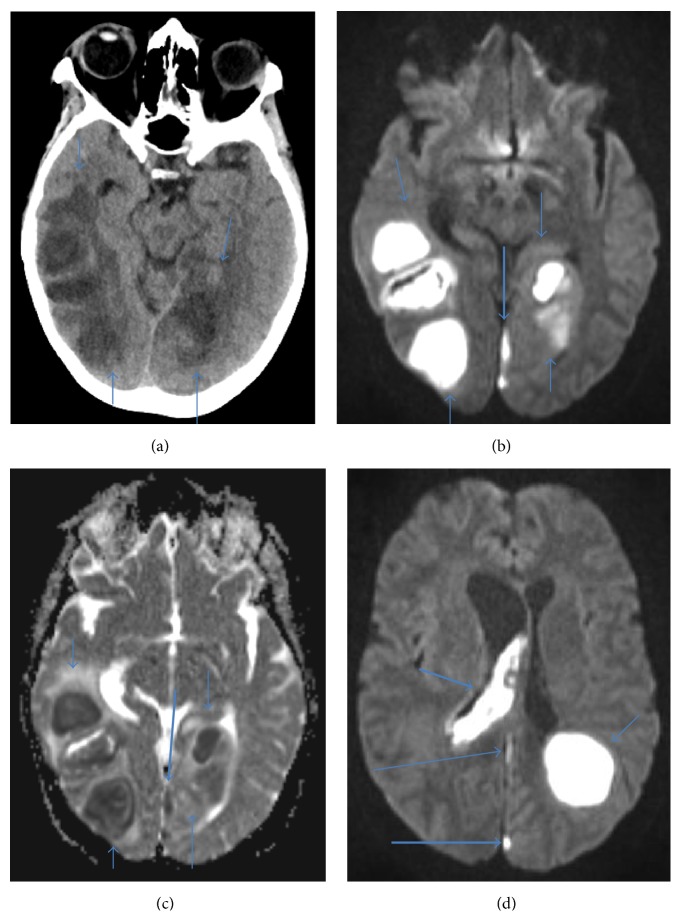
(a) Noncontrast axial CT image, (b) axial diffusion weighted images, (c) axial adjusted diffusion coefficient map that shows bilateral temporal-occipital lobe abscesses (thin arrows) and empyema in the medial left occipital lobe (thick arrow), and (d) axial diffusion weighted image that shows ventriculitis in the right lateral ventricle (short thick arrow), abscess in the left parietal lobe (short thin arrow), and empyema in the posterior falx (long thin arrow) and medial left occipital lobe (long thick arrow).

**Figure 2:**
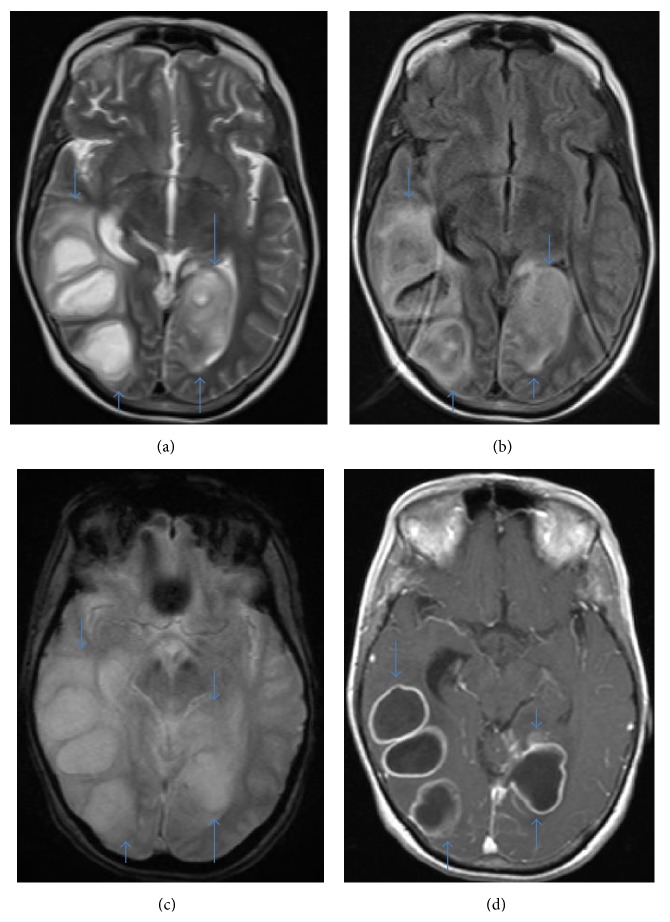
(a) Axial T2 weighted image, (b) axial FLAIR weighted image, (c) axial GRE weighted image, and (d) axial T1 weighted postcontrast image show bilateral temporal-occipital lobe abscesses (arrows).

CT and MRI with IV contrast showed 5 abscesses and periabscess edema (cf.  Table 2, Figures 1-2). CT understated the size of the abscesses probably because of bone beaming hardening artifact. However, the size of the edema surrounding the abscesses on CT and MRI is similar. On MRI, there was pus in the right lateral ventricle and 4th ventricle and posterior falx and medial left occipital lobe empyemas. There was edema and mass effect with a right to left midline shift of about 9 mm.

Patient underwent neurosurgery to drain the abscesses twice, first right cerebral abscesses, and then left cerebral abscesses. Patient had dural sinus thrombosis and was started on full dose heparin; she improved after surgery and treatment. She became hypotensive likely secondary to reinfection and developed large retroperitoneal hematoma which further exacerbated her shock. Poor prognosis was discussed with family and their decision to withdraw artificial means of survival was made. The patient died due to septic shock and brain abscesses secondary to* Streptococcus anginosus* despite aggressive antibiotic and surgical treatment.

## 3. Discussion

Brain abscess goes through four stages: early cerebritis, late cerebritis, early capsule, and late capsule. Cerebritis is acute inflammation consisting of neutrophils, macrophages, lymphocytes, and plasma cells with or without parenchymal necrosis. Abscess capsule is a vascular tissue lining, collagen, reactive gliosis, firm, and fibrous and contains lipid-laden macrophages, capillary proliferation, and chronic inflammatory cells [1].

Most abscesses are successfully treated with antibiotics. Surgical drainage may be required depending on the patient's clinical condition, the location of the abscess, and risk of intraventricular rupture (which carries a high mortality). Nearly 90% of abscesses are pyogenic and the mortality rate may be as high as 14% despite antibiotic treatment [5]. Brain abscess may successfully be treated by antibiotic treatment and surgical procedures, either aspiration or excision [6]. This is 61-year-old female with brain abscess that was promptly diagnosed and treated medically and surgically but died about 5 weeks after admission. According to Seydoux and Francioli [8], the mean delay between occurrence of first symptoms and hospitalization is significantly shorter for patients with poor outcome (death or severe sequelae) than for patients who recovered (fully or with moderate sequelae). Moreover, severely impaired mental status and neurological impairment at admission were associated with a poor outcome in terms of both mortality and sequelae. In all cases with fatal outcome or severe sequelae, the diagnosis was made and treatment was initiated within 24 hours of admission.

### 3.1. Presentation

This patient presented with altered mental status, confusion and agitation, and neurologic deficit. Symptoms of brain abscess depend on location, mass effect, and complications. Headache, fever, focal neurologic deficit, nausea, vomiting, neck stiffness, lethargy, hemiparesis, or seizures may be presenting symptoms of brain abscess. Laboratory tests may add little to the diagnosis of brain abscess [2, 5].

### 3.2. Imaging

Most abscesses are in the frontal and temporal lobes, normally at the gray-white matter junction. The MRI features of abscess are related to free radicals by phagocytic macrophages in the abscess capsule wall. Ring enhancement is not specific for pyogenic abscess and may be seen in other conditions such as nonpyogenic abscess, high grade neoplasm, primary central nervous system lymphoma, metastasis, infarct, hematoma, thrombosed giant aneurysm, radiation necrosis, demyelinating disease, and* Toxoplasmosis gondii* infection. Imaging characteristics of abscess are (1) 2–7 mm continuous smooth thin rim of enhancement, (2) T2 hyperintense rim, and (3) thinning along the medial wall. Abscess demonstrates high signal intensity on diffusion weighted image and low signal intensity of adjusted diffusion coefficient map. The sensitivity of diffusion weighted imaging in differentiation brain abscess from other brain lesions is 72–95% and 96–100%, respectively. A higher temperature around the abscess capsule than in the brain tissue more distant to the capsule, indicative of inflammation has been demonstrated by a thermosensitive MRI protocol [1, 2, 5, 8–16]. In this patient, there were abscesses in both temporal lobes, both occipital lobes, and left parietal lobe (cf.  Table 2).

The necrotic centers of bacterial abscess lack the normal brain metabolites of N-acetylaspartate (NAA), choline, and creatine. The typical resonances within the cavity of untreated pyogenic abscess cavity are elevated cytosolic amino acids (0.9 ppm) and lactate (1.3 ppm) with or without acetate (1.9 ppm) and succinate (2.4 ppm). Although lactate and lipid may be found in brain tumor and brain abscess, cytosolic amino acids (valine, leucine, and isoleucine) are only present in pyogenic brain abscess. Lactate, acetate, and succinate are the byproducts of glycolysis and fermentation by the causative bacteria whereas the amino acids are the results of proteolysis by polymorphonucleocytes in pus. The sensitivity and specificity of MR spectroscopy in distinguishing pyogenic brain abscess from other brain lesion are 72–96% and 30–100%, respectively. [1, 2, 5, 17–19].

Brain perfusion imaging demonstrates that the mean relative cerebral blood volume of brain tumor is significantly higher than that of brain abscess due to higher vascularity and blood brain barrier breakdown in the wall of tumor relative to the collagen wall of abscess. Diffusion tensor imaging shows reduced diffusivity and increased fractional anisotropy due to increased regulation of neuroinflammatory adhesion molecules causing a structure orientation of inflammatory cells in abscess cavity. The sensitivity of increased fractional anisotropy and reduced diffusivity in predicting pyogenic abscess is 100% and 75%, respectively [2].

### 3.3. Treatment

The size, location, number of abscess, and type of abscess influence the choice of treatment of brain abscess. Brain abscesses of about 2-3 cm are usually treated medically whilst brain abscesses larger than 3 cm or associated with mass effect are usually treated with stereotactic aspiration or excision combined with intravenous antibiotics. Most brain abscesses may be successfully treated with intravenous antibiotics alone for 6–8 weeks in about 10% of brain abscesses. Surgical aspiration/drainage and excision of brain abscess may be needed in about 75% and 15%, respectively, depending on the location of the brain abscess; risk of intraventricular rupture (which carries a high mortality), patient's clinical condition, when the abscess increases in size, and neurological problems develop; the abscess fails to decrease in size after about 3-4 weeks of antibiotic treatment, or recurrent large and superficial abscess despite multiple drainages. A routine follow-up CT or MRI every 2 weeks if new neurological signs develop may be required [2–5]. Nearly 90% of abscesses are pyogenic and the mortality rate may be as high despite antibiotic treatment [5]. The patient died due to septic shock despite aggressive antibiotic and surgical treatment.

### 3.4. Complications

Brain abscess complications include local mass effect with intracranial herniation, hydrocephalus, meningitis, extra-axial fluid collection, sinus thrombosis, infarct, cranial nerve involvement, ventriculitis, and extra-axial fluid collections (empyema and hygroma). This patient had mass effect, sinus thrombosis, empyemas, and ventriculitis. Intraventricular rupture with ventriculitis is a devastating complication associated with high mortality and appears as ventricular debris and abnormal ependymal enhancement. Empyemas have thicker enhancing rim than that of sterile effusions and nonspecific internal septations [2, 5, 20].

### 3.5. Prognosis

This is a 61-year-old female with brain abscess that was promptly diagnosed and treated medically and surgically but died about 5 weeks after admission. For brain abscess, the mean delay between occurrence of first symptoms and hospitalization is significantly shorter for patients with poor outcome (death or severe sequelae) than for patients who recovered (fully or with moderate sequelae). Severely impaired mental status and neurological impairment at admission are associated with a poor outcome in terms of both mortality and sequelae. In fatal outcome or severe sequelae, the diagnosis is made and treatment was initiated within 24 hours of admission [8]. Prognosis of brain abscess may be worse in multiple abscesses, intraventricular rupture of abscesses, and deeply located abscesses. The prognosis of brain abscess appears to be mainly determined by the rapidity of progression of the disease before hospitalization and the patient's mental status on admission [3, 8]. This patient had ventriculitis and poor mental status at presentation and expired about 5 weeks after admission despite aggressive medical and surgical interventions.

### 3.6. Predictive Value of Imaging Severity Index Score

To the best of my knowledge, the only imaging severity index score (ISI) is that proposed by Demir et al. [3] (Table 3) which had a better prognostic value than the Glasgow coma scale. Using an ISI score cut-off of 8, only 3 out of 41 patients with image severity index score of 8 or less demonstrated any adverse events whereas 38 of 55 patients having image severity index score of 9 or higher had disability. The image severity index score of all their patients that died was 10 or higher. This patient's image severity index score was 12; the patient died about 5 weeks after admission despite aggressive antibiotic and surgical treatments. Image severity index score which can be used by clinicians and radiologists is simple and reproducible and uses only five imaging parameters: number, location, size, amount of surrounding edema, and extent of midline shift. There is a recommendation for modification of the image severity index score to specifically take into account empyema and ventriculitis because these complications are associated with worse prognosis (Table 4).

### 3.7. *Streptococcus anginosus*

*S. anginosus* (milleri) group streptococci are the most common microbes associated with bacterial intracerebral abscesses. The* Streptococcus anginosus* group is made up of three species,* S. anginosus*,* S. constellatus*, and* S. intermedius*, and is part of viridans group of streptococci.* S. anginosus* group is normal flora in human oropharyngeal, gastrointestinal, and genitourinary tracts but are strongly associated with abscess formation or pyogenic invasive infections including multiple types of infection: head and neck (periodontitis, odontogenic abscesses, sinusitis, and pharyngitis), central nervous system (brain and spinal cord abscesses), pulmonary (lung parenchyma and pleural abscesses, and pneumonia), cardiovascular (endocarditis and bacteremia), and abdominal cavity (hepatic abscesses, gastrointestinal tract infections, peritoneal abscesses, cholangitis, and appendicitis), soft tissue, bone, and skin (cellulitis, osteomyelitis, subcutaneous and muscle abscesses, and wound infections). The ability of* S. anginosus* group bacteria to form abscesses may be due to its ability to inhibit lysis within neutrophils after phagocytosis. Although greater than 90% of the anginosus group streptococci are susceptible to penicillin, abscesses caused by these microbes may require surgical treatment [7, 21–25].

This patient had multiple abscesses in both temporal lobes, both occipital lobes, and left parietal lobe due to* Streptococcus anginosus* (cf.  Table 2). In a retrospective analysis of 49 cases of brain abscesses, (i)* S. anginosus* (milleri) group was the most commonly isolated microorganism in brain abscesses (11 of the 49 cases, 22%), and (ii) multiple organisms were isolated from 8 specimens out of 42 cases (19%) with majority containing Gram-positive cocci and Gram-negative rods [26].* Streptococcus anginosus* may cause hypothyroidism, meningitis, cerebral venous system thrombophlebitis, intracranial arteritis, inflammation and thrombosis of the cavernous sinus, and inflammation of the carotid sheath causing monoplegia and hemiplegia [27]. This patient had dural sinus thrombosis, was started on full dose heparin, became hypotensive likely secondary to reinfection, and developed large retroperitoneal hematoma which further exacerbated her shock. Direct specimen culture is the gold standard method of identifying the microorganisms causing* Streptococcus anginosus* brain abscess. A negative blood culture does not exclude the presence of live bacteria; blood cultures are negative in 24–20% of intracerebral abscesses [28, 29]. Patient underwent neurosurgery to drain the abscesses and* Streptococcus anginosus* was cultured from the purulent material evacuated from the abscess.

## Figures and Tables

**Table 1:** Imaging characteristics of abscess [1–20].

Stage	Description	Histology	MRI	MR spectroscopy	CT	CT perfusion
I	Early cerebritis, intermediate cerebritis	Early infection, inflammation. Poorly demarcated, toxic changes, and perivascular infiltrates	T1 isointensity to hypointensity. T2 hyperintensity without a defined margin; ill-defined nodular enhancement		Ill-defined low attenuation, variable contrast enhancement (no enhancement, nodular, or ring enhancement)	

II	Late cerebritis	Reticular matrix (collagen precursor), developing necrotic center	Increasing edema, development of rim of granulation tissue. T1 hypointensity. T2 isointensity to hyperintensity with surrounding edema; incomplete to complete zone of enhancement		Poorly defined low attenuation edema; thick ring or nodular enhancement	

III	Early capsule	Neovascularity, necrotic center, and periphery reticular matrix	Peripheral zone of enhancement is thinner, more uniform in contour, and relative to mesial thinning. No persistent central enhancement		Core is round or ovoid low attenuation, sometimes faint surrounding capsule ring. Ring enhancement corresponds to granulation tissue of capsule; medial or ventricular thinner than lateral wall due to differences in capsule blood supply	

IV	Late capsule	Collagen capsule, necrotic center, and gliosis around capsule	Loss of capsular hypointensity on T2-weighted images, reduction in size of central necrotic cavity. Enhancement may persist for several months, but progressively decreases on serial examination		Same as III above	
			Increased signal intensity of DWI, decreased signal intensity on ADC map	Metabolites identified in abscess: lactate, succinate, acetate, amino acids (valine, leucine, and isoleucine), and aspartate		Increase in cerebral blood flow, cerebral blood volume seen in 24 hours, and peak in 48–72 hours
			Increased fractional anisotropy and reduced mean diffusivity	Late capsule: necrotic center lack normal brain metabolites of NAA, choline, and creatine. Elevated cytosolic amino acids (valine, leucine, and isoleucine), lactate, acetate, and succinate		

**Table 2:** Brain abscesses by MRI and CT with IV contrast done on the same date.

Location	Transverse diameter (cm)		Anterior posterior diameter (cm)		Craniocaudal diameter (cm)		Periabscess edema (cm)	
	MRI	CT	MRI	CT	MRI	CT	MRI	CT
Right temporal lobe	2.6	2.3	2.3	1.8	3.5	N/A	1.3	1.3
Right temporal lobe	3.3	2.6	2.1	1.3	3.4	N/A	0.6	0.8
Right occipital lobe	2.7	2.6	2.9	2.4	3.0	N/A	0.8	0.8
Left occipital lobe	2.5	2.4	3.3	2.5	2.0	N/A	0.6	0.6
Left parietal lobe	3.1	2.4	3.3	2.5	3.2	N/A	1.1	1.3

**Table 3:** Image severity index score [3].

Parameters	Points
Number (single, multilocular, or attached to each other like a bunch of grapes = 1; score increased for every additional abscess)	
Solitary	1
Multiple	2–6

Location (superficial: cerebral, cerebellar hemispheres; deep: basal ganglia, thalamus, corpus callosum, brain stem, vermis, and within ventricles; extensive or combined: both superficial and deep)	
Superficial	1
Deep	2
Combined	3

Diameter (large diameter of abscess in transverse plane on CT and MRI; largest diameter of multilocular abscess is the largest diameter of the abscess taken as a whole)	
<2 cm	1
2–4 cm	2
>4 cm	3

Perilesional edema (surrounding edema observed as high signal intensity on T2 weighted MRI image or hypodensity on CT image. For multiple abscesses, highest for edema was used)	
Minimal (maximum thickness < radius of abscess)	1
Moderate (maximum thickness between the radius and diameter of abscess)	2
Large (maximum thickness > diameter of abscess)	3

Midline shift (in mm)	
Mild (<5 mm)	1
Moderate (5–10 mm)	2
Severe (>10 mm)	3

**Table 4:** Image severity index score [3] and proposed modifications in italic letters.

Parameters	Points
Number (single, multilocular, or attached to each other like a bunch of grapes = 1; score increased for every additional abscess)	
Solitary	1
Multiple	2–6

Location (superficial: cerebral, cerebellar hemispheres; deep: basal ganglia, thalamus, corpus callosum, brain stem, vermis, and within ventricles; extensive or combined: both superficial and deep)	
Superficial	1
Deep	2
*Empyema*	3
*Ventriculitis*	4
Combined	5

Diameter (large diameter of abscess in transverse plane on CT and MRI; largest diameter of multilocular abscess is the largest diameter of the abscess taken as a whole)	
<2 cm	1
2–4 cm	2
>4 cm	3

Perilesional edema (surrounding edema observed as high signal intensity on T2 weighted MRI image or hypodensity on CT image. For multiple abscesses, highest for edema was used)	
Minimal (maximum thickness < radius of abscess)	1
Moderate (maximum thickness between the radius and diameter of abscess)	2
Large (maximum thickness > diameter of abscess)	3

Midline shift (in mm)	
Mild (<5 mm)	1
Moderate (5–10 mm)	2
Severe (>10 mm)	3
